# New Treatments for Bacterial Keratitis

**DOI:** 10.1155/2012/831502

**Published:** 2012-09-06

**Authors:** Raymond L. M. Wong, R. A. Gangwani, Lester W. H. Yu, Jimmy S. M. Lai

**Affiliations:** ^1^Eye Institute, The University of Hong Kong, Room 301, Level 3, Block B, 100 Cyberport Road, Cyberport 4, Hong Kong; ^2^Department of Ophthalmology, Queen Mary Hospital, Hong Kong

## Abstract

*Purpose*. To review the newer treatments for bacterial keratitis. *Data Sources*. PubMed literature search up to April 2012. *Study Selection*. Key words used for literature search: “infectious keratitis”, “microbial keratitis”, “infective keratitis”, “new treatments for infectious keratitis”, “fourth generation fluoroquinolones”, “moxifloxacin”, “gatifloxacin”, “collagen cross-linking”, and “photodynamic therapy”. *Data Extraction*. Over 2400 articles were retrieved. Large scale studies or publications at more recent dates were selected. *Data Synthesis*. Broad spectrum antibiotics have been the main stay of treatment for bacterial keratitis but with the emergence of bacterial resistance; there is a need for newer antimicrobial agents and treatment methods. Fourth-generation fluoroquinolones and corneal collagen cross-linking are amongst the new treatments. In vitro studies and prospective clinical trials have shown that fourth-generation fluoroquinolones are better than the older generation fluoroquinolones and are as potent as combined fortified antibiotics against common pathogens that cause bacterial keratitis. Collagen cross-linking was shown to improve healing of infectious corneal ulcer in treatment-resistant cases or as an adjunct to antibiotics treatment. *Conclusion*. Fourth-generation fluoroquinolones are good alternatives to standard treatment of bacterial keratitis using combined fortified topical antibiotics. Collagen cross-linking may be considered in treatment-resistant infectious keratitis or as an adjunct to antibiotics therapy.

## 1. Introduction

Infectious keratitis is a potentially blinding ocular condition of cornea which can cause severe visual loss if not treated at early stage. If the appropriate antimicrobial treatment is delayed, only 50% of the eyes gain good visual recovery [1]. It can be caused by bacteria, virus, fungus, protozoa, and parasites. The common risk factors for infectious keratitis include ocular trauma, contact lens wear, recent ocular surgery, preexisting ocular surface disease, dry eyes, lid deformity, corneal sensational impairment, chronic use of topical steroids, and systemic immunosuppression [2–5]. The common pathogens include *Staphylococcus aureus*, *coagulase-negative Staphylococcus*, *Pseudomonas aeruginosa*, *Streptococcus pneumonia,* and *Serratia* species. The majority of community acquired cases of bacterial keratitis resolve with empiric treatment and do not require culture [6]. Corneal scraping for culture and sensitivity is indicated for corneal ulcers that are large in size, central in location, extend from middle to deep stroma, associated with pain, simultaneous presence of anterior chamber reaction or hypopyon, poor vision, and presence of corneal abscess or unresponsive to broad spectrum antibiotic therapy [6]. Recent studies have shown increasing evidence of resistance of microbes to antimicrobial agents [7–9]. Microorganisms develop resistance due to chromosomal mutation, expression of latent chromosomal genes by induction or exchange of genetic material via transformation [9, 10]. This can cause continued progression of the disease process despite the use of broad spectrum antibiotics. The purpose of this study was to review the newer treatments available for treating the infectious keratitis including those which are resistant to the antimicrobial therapy.

## 2. Methods

A PubMed literature search was conducted up to April 2012 using the following key words: “infectious keratitis”, “microbial keratitis”, “infective keratitis”, “new treatments for infectious keratitis”, “fourth generation fluoroquinolones”, “moxifloxacin”, “gatifloxacin”, “collagen cross-linking”, and “photodynamic therapy”. Articles reporting the efficacies of using fourth-generation fluoroquinolones or photodynamic therapy in the treatment of infectious keratitis were selected and analyzed. During selection of the articles, prospective studies had a higher ranking than the retrospective studies, and clinical/in vivo studies had higher ranking than in vitro studies. 

## 3. Highlights of Literature Review

### 3.1. Infectious Keratitis

Corneal ulcer or infectious keratitis is a serious condition of cornea that requires prompt management. When a patient presents with the features of infectious keratitis, clinical history and detailed clinical examination guide to the category of high risk or low risk characteristics [3]. Presence of history of ocular trauma, contact lens use, preexisting ocular surface disease, history of long term or injudicious use of topical steroids, large size of ulcer, and central location of ulcer are considered to be high risk characteristics. According to the American Academy of Ophthalmology guidelines for bacterial keratitis, most of the cases of community acquired infectious keratitis respond to the empirical treatment with antibiotics. Corneal scraping is indicated for corneal ulcers that are large in size, central in location, extend from middle to deep stroma, associated with pain, simultaneous presence of anterior chamber reaction or hypopyon, poor vision, and presence of corneal abscess or unresponsive to broad spectrum antibiotic therapy [6]. The culture-guided approach consists of taking a sample of corneal tissue by corneal scraping or biopsy and performing microbiological tests to determine the type of bacterial organisms and their sensitivity to the particular group of antibiotics. However, empirical antibiotics will usually be started after microbiological specimens have been collected if there is clinical suspicion of infection.

## 4. Treatment Options

### 4.1. Fluoroquinolones

Fluoroquinolones are synthetic broad spectrum antibiotics. They inhibit DNA gyrase (topoisomerase II) and topoisomerase IV enzyme, which are key enzymes involved in DNA replication and transcription [11]. Inhibition of these enzymes will lead to bacterial cell death [12]. Topoisomerase IV is the main target for most Gram-positive bacteria. DNA gyrase on the other hand is the main target for Gram-negative bacteria [12]. Nalidixic acid, the first generation fluoroquinolone, was used to treat urinary tract infection. The increasing incidence of resistance to earlier generation fluoroquinolones pointed to the need of newer generation antibiotics [13, 14]. The second-generation fluoroquinolones include ciprofloxacin and ofloxacin; third-generation fluoroquinolones include levofloxacin, fourth-generation fluoroquinolones include moxifloxacin and gatifloxacin. The advances in molecular structures of fourth-generation fluoroquinolones, that is, moxifloxacin and gatifloxacin, resulted in inhibition of both DNA gyrase and topoisomerase IV in Gram-positive bacteria [15]. These changes increase the antibiotic potency against Gram-positive organisms while maintaining their broad spectrum activities against Gram-negative bacteria [12]. These structural modifications also reduce the risk of development of resistant organisms since two concomitant mutations are necessary for the development of resistance [16–18]. Furthermore, the structure of moxifloxacin is resistant to bacterial cells' efflux mechanism, thus enhancing its potency to kill bacteria [11]. Ophthalmic application of fluoroquinolones began in the 1990s when the second generation fluoroquinolones like ciprofloxacin and ofloxacin were available in topical form. They were used for the treatment of infectious keratitis and conjunctivitis [19, 20]. In this paper, we reviewed the literature and looked into the clinical use of fourth-generation fluoroquinolones in the treatment of infectious keratitis.

#### 4.1.1. In Vitro Potency of Fluoroquinolones

The potency of antibiotics against bacteria is reflected by the minimum inhibitory concentration (MIC) obtained for different organisms during microbiological analysis. A drug with a low MIC for a particular organism means that it has a potent antibiotic effect on this particular organism. Kowalski et al. determined the MIC90s of 177 bacterial keratitis isolates to ciprofloxacin, ofloxacin, levofloxacin, gatifloxacin, and moxifloxacin [21]. They found that the MIC90s for Gram-positive bacteria were significantly lower for fourth-generation fluoroquinolones than second- or third-generations, especially for fluoroquinolone-resistant *Staphylococcus aureus* (3.0 ug/mL in moxifloxacin and gatifloxacin versus 64.0 ug/mL in levofloxacin, ciprofloxacin, and ofloxacin). However, ciprofloxacin (2nd generation) is still better than the third-and fourth-generation fluoroquinolones against Gram-negative organisms including *Pseudomonas aeruginosa* (ciprofloxacin 0.125 ug/mL, ofloxacin 1.5 ug/mL, levofloxacin 0.5 ug/mL, moxifloxacin 0.75 ug/mL, gatifloxacin 0.38 ug/mL). Among the two fourth-generation fluoroquinolones, moxifloxacin demonstrated statistically lower MIC90s for most Gram-positive bacteria; gatifloxacin on the other hand was noted to have lower MIC90s for most Gram-negative bacteria [21]. Sueke et al. collected 772 bacterial isolates from cases of bacterial keratitis in multiple centers in the United Kingdom and tested against standard and new antibiotics [22]. Among the fluoroquinolones (ciprofloxacin, ofloxacin, levofloxacin, and moxifloxacin), moxifloxacin demonstrated the lowest MICs for both Gram-positive and Gram-negative bacteria [22]. Chawla et al. identified 292 bacterial isolates from consecutive cases of suspected bacterial keratitis and reviewed their microbiological response to cefazolin, tobramycin, gatifloxacin, and moxifloxacin [23]. Susceptibilities to moxifloxacin and gatifloxacin were similar: 92.8% and 95.5% of all the bacterial isolates were susceptible to moxifloxacin and gatifloxacin, respectively. Only 83.6% and 90.1% of the isolates were susceptible to cefazolin and tobramycin, respectively [23]. A few other studies have tried to look into the in vitro susceptibilities of bacterial isolates obtained from ocular infections such as blepharitis, conjunctivitis, keratitis, and endophthalmitis to the commonly prescribed antibiotics. Similar results regarding fluoroquinolones were obtained in these studies in which the fourth-generation fluoroquinolones were generally superior to other generations of fluoroquinolones in their actions on Gram-positive bacteria [24–27]. Though consistent results were obtained for Gram-positive organisms among these studies, in the study by Oliveira et al., ciprofloxacin had lower MICs than the two fourth-generation fluoroquinolones for Gram-negative bacteria, especially for *Pseudomonas* species [27]. The results of in vitro studies may not be directly translated to clinical effectiveness because there are no susceptibility breakpoints for topically applied antibiotics to the eye.

#### 4.1.2. Clinical Trials on Fluoroquinolones

Three clinical trials were found in the literature investigating on the clinical efficacy of the fourth-generation fluoroquinolones in treating infectious keratitis. The largest study was conducted by Constantinou et al. [28]. They recruited 229 patients with bacterial keratitis and randomized to three treatment groups, the moxifloxacin (1.0%) group, the ofloxacin (0.3%) group, and the combined fortified tobramycin (1.33%)/cefazolin (5.0%) group. All the patients were given hourly instillation of the topical antibiotics in the first 48 hours then tapered off according to the protocol until after the 7th day when frequency of instillation will be adjusted according to clinical response. Of the bacterial isolates obtained, none of them were resistant to moxifloxacin, 2.5% were resistant to ofloxacin, 2.8% to ciprofloxacin, 14.8% to cefazolin, 1.6% to tobramycin, and 17.5% to chloramphenicol. The cure rate, the mean time to cure, the clinical sign score, and the rate of serious complications were not significantly different among the three groups. Two patients reported stinging and one developed ulceration of the inferior bulbar conjunctiva after applying antibiotics eyedrops, all of them were from the fortified treatment group. None of these minor complications were noted in the fluoroquinolones monotherapy groups. Another study conducted by Parmar et al. compared the effect of topical gatifloxacin 0.3%, a fourth-generation fluoroquinolone, with ciprofloxacin 0.3%, a second-generation fluoroquinolone, for the treatment of patient with bacterial keratitis and ulcer size of at least 2 mm [29]. This study recruited a total of 104 patients randomized to the two treatment group, with in-patient hourly instillation of topical antibiotics until the ulcer began to heal with dosing frequencies adjusted accordingly. Culture results revealed that significantly larger proportion of both Gram-positive and Gram-negative bacteria were susceptible to gatifloxacin than ciprofloxacin. 96.2% of Gram-positive cocci were susceptible to gatifloxacin versus 60.4% to ciprofloxacin; all Gram-positive bacilli were susceptible to gatifloxacin but only 75% were susceptible to ciprofloxacin; 92.9% of Gram-negative bacilli were susceptible to gatifloxacin compared to 85.7% to ciprofloxacin. Even for *Pseudomonas aeruginosa*, 87.5% were susceptible to gatifloxacin while only 75% were susceptible to ciprofloxacin. Clinically, 95.1% of patients in gatifloxacin group enjoyed good response and complete healing of ulcer, which was significantly higher than the ciprofloxacin group in which only 80.9% of patients had complete healing. The mean time taken for the ulcer to heal was similar in the two groups. The latest clinical study of these kinds was conducted by Shah et al. in 2010 [30]. A total of 61 patients were randomized to three groups comparing the clinical effects of moxifloxacin 0.5%, gatifloxacin 0.5%, and combined fortified tobramycin 1.3%/cefazolin 5% on bacterial keratitis. All the patients suffered clinically from bacterial keratitis with ulcer size between 2 mm and 8 mm. In this study, 46% of the subjects had eye injury before the episode. Topical antibiotics were instilled hourly for the first 48–72 hours and then tapered off according to the study protocol. Of the bacterial isolates tested, 5.2% were resistant to tobramycin and 10.4% were resistant to cefazolin. All isolates were susceptible to the two 4th generation fluoroquinolones under study. The cure rates of the fortified antibiotics group was 90% and of the gatifloxacin and moxifloxacin group 95%. However, the difference was not statistically significant. The mean duration to heal, the final visual acuity, and the size of the corneal opacities at the end of the study were also found to be statistically insignificant. Two patients complained of mild ocular discomfort after applying gatifloxacin. No other adverse effect was reported.

### 4.2. Collagen Cross-Linking (CXL)

Approximately 90% of corneal thickness is composed of stroma. Corneal stroma is made up of regularly arranged collagen fibrils with presence of keratocytes. Bacteria and fungi produce enzymes which have ability to digest human collagen and cause corneal melting. 

Collagen cross-linking (CXL) is a technique that uses riboflavin and Ultraviolet-A irradiation to cause a strengthening effect in corneal tissue which enhances its rigidity [31–33]. The interactive effect of riboflavin with UV-A irradiation strengthens formation of chemical bonds between collagen fibrils in the corneal stroma and helps in increasing resistance against enzymatic digestion [34]. Riboflavin or vitamin B2 is a naturally occurring substance. It is an important micronutrient which plays a key role in maintaining health in human beings. It was demonstrated by Japanese scientists that when riboflavin was exposed to visible or UV light, it could be used to inactivate the RNA containing tobacco mosaic virus [35]. Since that discovery, this phenomenon has been used in several subspecialties of medicine to inactivate viruses, bacteria, and parasites [36–39]. The photoactivation of riboflavin causes damage to RNA and DNA of microorganisms by oxidation processes and causes lesions in the chromosomal strands [13]. In addition, the ultraviolet irradiation itself has sporicidal and virucidal effects [40, 41]. 

The procedures of collagen cross-linking used in the treatment of infectious keratitis are almost identical to the standard protocol of treatment of keratoconus, with the exception that after application of anaesthetic eyedrops, only loose epithelium and the epithelium around the infectious site were removed in infectious keratitis [42–44]. The purpose of removing the corneal epithelium is to achieve adequate penetration of riboflavin eye drops. Riboflavin (riboflavin/dextran solution 0.5–0.1%) is instilled over the surface of cornea for a period of 20–30 minutes at an interval of 2-3 minutes. This is followed by illumination of the cornea using a UV-X lamp, UV-A 365 nm, with an irradiance of 3.0 mW/cm^2^ and total dose of 5.4 J/cm^2^. 

#### 4.2.1. In Vitro Studies of CXL

Spoerl et al. showed that cross-linked corneas had increased resistance against enzymatic digestion by proteinases and collagenase [45]. Martins et al. conducted an in vitro study to demonstrate the antimicrobial properties of riboflavin/UVA (365 nm) against common pathogens. They found this treatment to be effective against certain bacteria such as *Staphylococcus aureus* (SA), *Staphylococcus epidermidis* (SE), methicillin-resistant *S. aureus* (MRSA), *Pseudomonas aeruginosa*, and drug-resistant *Streptococcus pneumoniae* but ineffective against* Candida albicans *[46]. In a study by Kashiwabuchi et al., the authors did not find treatment with UVA + riboflavin to be effective against trophozoites of *Acanthamoeba* in vitro or in vivo [47]. Despite being ineffective in the in vitro test, in a case report by GarduñoVieyra et al. and, case series by Khan et al. YA, UVA + riboflavin was shown to be effective in the treatment of *Acanthamoeba* keratitis. Their patients showed rapid reduction in ocular symptoms and ulcer size [48, 49]. 

#### 4.2.2. Clinical Studies

Corneal CXL was initially used in conditions of corneal ectasia, for example, keratoconus. Collagen CXL increases the biomechanical strength of cornea and helps in halting the progression of keratoconus [43, 50]. Müller et al. showed that CXL was able to improve healing in patients with corneal melting secondary to contact lens-related infectious keratitis [51]. Iseli et al. in their case series of 5 patients with antibiotics treatment-resistant infectious keratitis demonstrated the efficacy of UVA/riboflavin treatment in halting the progression of corneal melting [44]. In a study by Makdoumi et al. which consisted of 7 eyes, corneal melting was arrested and complete epithelialization achieved in all cases after collagen cross-linking treatment with riboflavin [42]. For the two patients presented with hypopyon, the hypopyon regressed two days after CXL [42]. In the most recent study by Makdoumi et al., CXL has been successfully used as the primary treatment in the subjects with infectious keratitis [52]. Only 2 out of the 16 patients in the study required antibiotics; one required amniotic membrane transplantation. Ferrari et al. also reported a case of Escherichia coli keratitis with no improvement with topical and systemic antibiotics but started to heal after CXL was used [53].

## 5. Discussion

The in vitro studies of the MIC of different antibiotics against keratitis isolates have provided an idea of the potencies of the fourth-generation fluoroquinolones moxifloxacin, gatifloxacin, and tobramycin-cefazolin against pathogens for infectious keratitis [23]. However, we cannot compare their relative potencies because they belong to different classes of antibiotics (fluoroquinolones, aminoglycosides, cephalosporins) which possess different mechanisms of action. Potency comparisons can only be made within the same class of antibiotics. The fourth-generation fluoroquinolones are found to be either similar to or better than earlier generations fluoroquinolones (e.g., ciprofloxacin, ofloxacin, levofloxacin) in killing causative bacteria in infectious corneal ulcer [21, 22, 24–47]. Generally, moxifloxacin and gatifloxacin have higher potencies against Gram-positive organisms while maintaining its broad-spectrum activities against Gram-negative organisms. However, ciprofloxacin is still better than the third- and fourth-generation fluoroquinolones against Gram-negative bacteria including *Pseudomonas aeruginosa *[21]. As discussed, in vitro potency may not translate directly to clinical efficacy because the latter is also affected by the tissue penetration and the final tissue concentration of the antibiotics. However, since topical antibiotic eye drops in ocular tissues can usually achieve ten- to hundred-folds higher concentrations than the usual MIC for organisms [54, 55], even if a bacterial species is found to be resistant to a particular antibiotic in vitro, clinically it may still respond to that antibiotic. Also, moxifloxacin has an advantage over other fluoroquinolones such as gatifloxacin and levofloxacin in that it is able to achieve higher conjunctival, corneal, and aqueous concentrations [56–60]. MIC was also noted to be correlated to the corneal scar size after healing of infectious keratitis. For every two-fold increase in MIC, there will be a 0.33 mm increase in the diameter of the scar, though it is not found to be correlated with the best corrected visual acuity [61]. Therefore, the lower MICs of the fourth-generation fluoroquinolones shown in the in vitro studies imply potentially better healing of the corneal ulcer [21–27]. The results of the three clinical trials correlate well with the results of the in vitro studies in that fourth-generation fluoroquinolones were comparable to fortified antibiotics and were better than the second-generation fluoroquinolones in the treatment of infectious keratitis [28–30]. The antibiotic resistance rates of the bacterial isolates were consistently lower in moxifloxacin and gatifloxacin than almost all other antibiotics. However, it is important to note that in the study by Constantinou et al. [28], the percentage of Gram-positive bacteria constituted 76.2% of all the bacterial isolates and Gram-negative bacteria constituted 23.8%. In contrast, the Hong Kong and the United Kingdom studies reported a different spectrum of pathogens in bacterial keratitis, with 46.8% Gram-positive and 53.2% Gram-negative in Hong Kong, [60] and 38.9% Gram-positive and 61.1% Gram-negative in the United Kingdom [62]. Since fourth-generation fluoroquinolones are known to have higher potency against Gram-positive bacteria [12] and lower potency than ciprofloxacin in the inhibition of *Pseudomonas aeruginosa *[21, 27], the comparable efficacies of moxifloxacin, ofloxacin, and combined fortified tobramycin/cefazolin may not be reproducible in countries like Hong Kong and the United Kingdom, especially when *Pseudomonas aeruginosa* only constituted 7% of all the isolates in Constantinou's study but 36.4% and 49.1% in Hong Kong and the United Kingdom. The cure rate by moxifloxacin could possibly be lower than that reported by Constantinou et al. This argument can also apply to the studies conducted by Parmar et al. (81.3% Gram-positive and 18.7% Gram-negative; 10.7% were *Pseudomonas aeruginosa*) [29] and Shah et al. (culture positive cases: 85.5% Gram-positive and 14.5% Gram-negative; 11.3% were *Pseudomonas aeruginosa*) [30]. Moreover, though the treatment failure rates were not significantly different, we can see that the actual percentage of treatment failure was lower in the fortified tobramycin/cefazolin group (0.0%) than the moxifloxacin group (10.6%) and the ofloxacin group (6.6%) [28]. Again, though not statistically significant, the mean durations to cure were shorter in the fortified tobramycin/cefazolin group (38.2 days) and moxifloxacin group (36.4 days) when compared with the ofloxacin group (46.2 days) [28]. Parmar et al. also reported that despite statistically insignificant, only 50% (1 out of 2) of the patients in the gatifloxacin group with *Pseudomonas* keratitis healed compared to 100% (5 out of 5) in the ciprofloxacin group [29]. This suggests that gatifloxacin may be less effective than ciprofloxacin against *Pseudomonas aeruginosa*. By sample size calculation based on an estimated difference of 15% among groups, >85% of overall response rate and 85% probability of detecting a difference, clinical studies need 77 subjects for each treatment group (i.e., 154 for studies involving two intervention groups and 231 for studies involving three intervention groups) in order to achieve sufficient power to identify the clinical differences concerned. Since the number of patients was too small in the study conducted by Shah et al. (around 20 patients per group) [30] and Parmar et al. (around 50 patients per group) [29], their results may not be able to reach a statistically significant level despite that a genuine difference exists. Thus, the data of these studies should be interpreted carefully and may only be seen as a supplement to other larger studies. The minimally invasive technique of CXL initially used in the management of ectatic conditions of cornea such as keratoconus, pellucid marginal degeneration, and iatrogenic keratectasia following laser in situ keratomileusis (LASIK) has been effectively used for treatment of infectious keratitis with or without the risk of corneal melting. Recent study has demonstrated the efficacy of this treatment modality in the primary treatment of infectious keratitis [52].

## 6. Conclusion

Topical fourth-generation fluoroquinolones, namely, moxifloxacin and gatifloxacin, are good alternatives to combination of fortified antibiotics in the management of infectious keratitis. They may be used as empirical therapies after corneal scraping has been performed. Low antibiotics resistances to these two fluoroquinolones are expected in view of their structural modifications and dual inhibition mechanisms. However, since moxifloxacin and gatifloxacin may not be as potent as ciprofloxacin or tobramycin against Gram-negative organisms such as *Pseudomonas aeruginosa,* further studies are warranted to compare the response of *Pseudomonas* infections to these antibiotics before we can conclude that the new fluoroquinolones are as potent as the standard combination of fortified antibiotics in the management of infectious keratitis. To date, only a few papers in the literature have reported the effect of photodynamic therapy (collagen CXL) in the management of infectious keratitis. The results of these trials are promising and imply that this new treatment modality may be useful in the treatment of resistant infectious corneal ulcer or as an adjunct for standard antibiotic treatment. However, since all of the published studies regarding CXL as the treatment of infectious keratitis were either based on animals or small numbers of patients, larger scale randomized, controlled trials should be conducted to evaluate the additional beneficial effects of CXL in infectious keratitis on top of conventional topical antibiotics. Furthermore, more evidence is required before it will be advisable to use CXL as the first line treatment for infectious corneal ulcers.
